# Mobile Health in Maternal and Newborn Care: Fuzzy Logic

**DOI:** 10.3390/ijerph110606494

**Published:** 2014-06-20

**Authors:** Shahirose Premji

**Affiliations:** 1 Faculty of Nursing, University of Calgary, 2500 University Drive NW, Calgary, AB T2N 1N4, Canada; 2 Faculty of Medicine, Department of Community Health Sciences, University of Calgary, 2500 University Drive NW, Calgary, AB T2N 1N4, Canada

**Keywords:** mobile phones, interpersonal relationships, social influence, mother-infant interaction, unintended harm

## Abstract

Whether mHealth improves maternal and newborn health outcomes remains uncertain as the response is perhaps not true or false but lies somewhere in between when considering unintended harmful consequences. Fuzzy logic, a mathematical approach to computing, extends the traditional binary “true or false” (one or zero) to exemplify this notion of partial truths that lies between completely true and false. The commentary explores health, socio-ecological and environmental consequences–positive, neutral or negative. Of particular significance is the negative influence of mHealth on maternal care-behaviors, which can increase stress reactivity and vulnerability to stress-induced illness across the lifespan of the child and establish pathways for intergenerational transmission of behaviors. A mHealth “fingerprinting” approach is essential to monitor psychosocial, economic, cultural, environmental and physical impact of mHealth intervention and make evidence-informed decision(s) about use of mHealth in maternal and newborn care.

## 1. Introduction

Mobile devices and other wireless devices and sensors are an attractive technology for delivering health care services and health promotion messages, collectively referred to as mHealth, as it is pervasive around the world in all cultural groups, economic levels and ages [1,2,3]. The world population, comprising those living in rural areas as well, has access to mobile networks. The international telecommunication union estimated 5.3 billion mobile subscriptions by end of 2010 with rates of subscription plateauing in high-income countries (1.6%) and increasing to 73% in low- and middle-income countries [4]. Extending the reach of the health care system, mHealth is intended to serve as “cue to action” to promote health care behavior change [1], thereby replacing the need for face-to-face interventions delivered at health facilities or at home.

MHealth’s interactive two-way communication strategies [5] generally entails short message service (SMS) text messaging to deliver primary care, provide family planning services, promote health, direct people to emergency care services, remind patients about follow-up appointments, collect health information, and promote and support exclusive breastfeeding [6,7]. MHealth is gaining global popularity across maternal and newborn care, including low- and middle-income countries (e.g., India, Kenya, Peru, South Africa, Tanzania) where mHealth is seen to potentially help meet targets for Millennium Development Goals 4 to 6 (*i.e.*, reduce child mortality, improve maternal health, and combat diseases such as malaria, HIV/AIDS) established during the Millennium Summit of the United Nations in 2000 [7,8]. In general, mHealth has many positive health benefits: improved access to medical services; promoted behavior change (e.g., smoking cessation, diabetes self-management, medication adherence); reduced no-shows at follow-up appointments; enhanced monitoring and management of diseases such as diabetes; improved data collection, management, and tracking (e.g., immunization records, prenatal care schedules); and enhanced teaching and training of both health care providers and patients [5,6,7,9,10].

Although these findings demonstrate promise for mHealth use in improving maternal and newborn health, the evidence informing these strategies lags behind [7,10], more so with respect to unintended harmful consequences characterized as psychosocial, economic, cultural, environmental, and physical [11]. This commentary addresses the socio-ecological and environmental consequences of mHealth [1], emphasizing the postpartum period (*i.e.*, after the birth of the baby), demonstrating mHealth may be a mixed blessing.

## 2. Unintended Harmful Consequences of mHealth

### 2.1. Psychosocial

#### 2.1.1. Mother-Infant Interactions

The ever-present nature of mobile communication, and that of mHealth, has implications for the behavioral and social dimensions of mothers’ relationship with their infants, particularly when considering dosage (*i.e.*, frequency of SMS text messaging) of mHealth. Mobile communication affects the nature of social interactions described as psychological “emptying out” [12,13]. The term “emptying out” as a condition is a sense of distant encounters taking priority over immediate social relations. The condition is also referred to as “absent presence” given the lack of awareness of the social surrounding [12,13]. Audio/video technology, such as commercially available programs specifically directed at infants such as infant-directed video, can alter mother-infant interactions [14]. For example, parents may talk to their infants less when a video is turned on compared to when it is turned off [14]. The social environment in early life profoundly influences the language, cognitive skills, and emotional competencies of babies given the brain’s plasticity in brain organization [14,15]. These competencies have lifelong benefits not only for growing children but also for society, as they help individuals become contributing members of society from both a social and economic standpoint. Early brain development has been linked to population well-being and adaptations to social changes, essential factors to promote human and social capital [15]. Impoverished mother-infant interactions (e.g., insensitive, lacks mutuality, emotionally unresponsive) in day-to-day child rearing can negatively influence physical, socio-emotional, and language/cognitive development of infants [15,16,17]. Early negative life events can increase stress reactivity and vulnerability to stress-induced illness across the lifespan of the individual [15,18]. Moreover, offspring may manifest similar patterns of maternal care behaviors; thus, neurobiological basis of these behaviors has implications for future generations (*i.e.*, intergenerational transmission of behaviors) [17,18,19].

Premature infants are likely to suffer the most from the unintended negative consequence of “emptying out” or “absent presence” of their mothers as a result of mHealth [12,13]. Premature infants need their mothers to be more sensitive to their movements, expressions, color, so as to adjust caregiving to their special needs. Premature infants, by virtue of their biologic make-up (e.g., shut-down quickly, do not give proper cues), are particularly difficult to engage with and influence the affective state of the caregiver perpetuating the cycle of diminished maternal sensitivity to infant needs [15]. This exemplifies the gene environment interaction in that the infant’s behavior, which is under genetic influence, increases the likelihood of exposure to negative environment (*i.e.*, evocative gene-environment correlation) [15]. Globally, 14 million babies survive premature birth every year [20]; their chance for developmental and behavioral success depends on the sensitive, responsive care they receive from caregivers.

#### 2.1.2. Mother-Provider Interactions

Although intended as a communication tool, mobile devices have significantly influenced our social relationships and promoted greater social cohesion [2,21]. mHealth can transform when, where, and how health care professional provide care to mothers and their infants [2]. With mobile communications, health care providers have reported feeling “close to the villagers” because the reduced social distance enabled them to build and maintain relationships with the community [22]. The “connected presence” refers to the qualities of connecting or feeling connected on an ongoing basis with others through mobile communication [23]. These interactions may expand mothers’ social networks by enhancing engagement (social and professional) between the health care provider and the community (*i.e.*, mothers) [22,24]. Paradoxically, these interactions may not necessarily deepen social relations [24] as technical accessibility to a health care provider does not equate to social availability, which is negotiated more so by the receiver [25]. Moreover, technical accessibility to a health care provider does not necessarily equate to accessibility to a health care professional who has the appropriate knowledge, skills, and attributes (*i.e.*, competencies) to address health care concerns [24]. It is important to appreciate that some mothers may have a preference for face-to-face communication [24].

Use of mobile technology has been shown to influence the sender, receiver and the quality of their collaborative relationship [26]. Technology-mediated interactions are facilitated by technological literacy (*i.e.*, “New literacy”) of both health care providers and recipients of care (*i.e.*, mothers) [22]. Unfamiliarity or lack of skills with mobile technology may pose significant barriers for health care providers and mothers to engage in a collaborative relationship. Temporal patterns have also been observed with use of SMS text by health care providers, which has implications for workflow design such as coordination of patient care [27]. Poor patterns of communication (e.g., frequency of interruptions with SMS text, text replies with inadequate information, time lag in responding) can potentially erode the collaborative relationship between the mother and health care provider. Frequency of interruption by multiple channels of communication (*i.e.*, direct calls and text messages) may disrupt workflow of the health care provider particularly in situations where the interaction cannot be differed. Time commitment required to make contact with the sender and determining the nature of the issue and action required may impose additional burden on the health care provider [26]. SMS text is useful for short, simple, conversational exchanges while more complex discussions require telephone or face-to-face conversations.

Establishing a professional relationship involves communicative and mental processes. Established rules and norms that are culturally specific guide interpersonal communication during which complex negotiations occur to create shared goals and shape identity [23]. In contrast, human-technology-human interactions have no established rules; rather rules are developed as the relationship evolves. There may be more than 130 messages sent when promoting postpartum care [1], which may increase social attachment between mothers and health care providers beyond the actual communication [1]. Consequently, mHealth may increase mothers’ dependency on health care provider vis-à-vis the mobile device, reducing her self-efficacy and confidence in providing care to her newborn.

Mobile devices are a double-edged sword in that they have made life more convenient yet more complex, evoking “electronic emotions” defined as “emotions that are lived, conveyed or created via electronically mediated communications” [28]. How these emotions manifest, given the human-like characteristics attributed to mobile technology, remain elusive [28], particularly when considering the labile nature of a woman’s emotions secondary to hormonal changes in the postpartum period [29]. When a mother “unplugs” from the mHealth program—the mHealth program concludes and the mother disconnects from the technology [30]—her inability to remain engaged may influence her perceived support and increase maternal anxiety, factors that increase her risk of postpartum depression [31]. Stress, anxiety and depression in the postpartum period may adversely influence infant survival, behavior, and development by way of poor quality of maternal-infant interactions and the stress load has implications for risk of preterm birth in future pregnancies [32,33,34,35,36].

The entrepreneurial nature of mobile technology [2], and perhaps health care, will provide additional impetus to move beyond sending text messages to encourage mothers to participate in improving their health outcomes and that of their infant. Advances in wireless application and development of data communication will spur the launch of smartphones and tablets loaded with health apps—the new wave of mHealth. Consequently, one can anticipate that mHealth will influence the socialization between health care providers and mothers in more profound ways than anticipated. Empirical research, therefore, must examine the impact of mHealth on the emotional and cognitive processes of mothers (e.g., stress, anxiety, depression, self-efficacy, maternal confidence in care) and communication practices between health care professionals and mothers [23]. The interrelationship between psychosocial, social, and economic consequences exemplifies the far-reaching potential negative consequences of mHealth.

### 2.2. Economic

The rise in mobile subscriptions in lower-income countries has been attributed to increased affordability from competition in the private sector [37]. In low- and middle-income countries mobile phone expenses account for approximately 2% of monthly expenditures [38]. The cost of acquiring and maintaining a mobile phone, however, may be prohibitive for health care providers [22] and for mothers when considering the average family income and other household expenditures [37]. Sustainability of mHealth has been questioned, particularly when programs have been subsidized [22]. In Aceh Besar, Indonesia, midwives who received monthly call credits to monitor and consult with their patients (*i.e.*, research study intervention group) expressed concern about the “financial burden” of purchasing phone credits when the monthly supply was low and lack of affordability if they had to purchase the mobile technology on their own [22]. Moreover, mHealth strategy increased the midwives’ workload whereas they received the same monthly salary with or without using mobile phones to communicate with their patients [22]. An increase in patient-load however was counterbalanced with greater work efficiency (e.g., limit number of home visits that are time consuming) [22].

Economic consideration also includes having a well-established mobile infrastructure, power generation and supply, and efficient use of power sources. Poor mobile network coverage can hinder health care professionals’ ability to communicate with mothers and with other health care providers [39]. The battery capacity of mobile devices is restricted to the dimensions of the devise (*i.e.*, size and weight), hence the battery has to be recharged frequently. Limited or erratic access to a power supply can hinder utilization of mobile devices for mHealth interventions, and as a result, mHealth programs have started investing in generators or mini solar chargers; the latter has variable efficacy [39,40]. Health care workers have needed supervision to: (a) ensure their mobile devices have enough power before they start work, and (b) regulate use of their mobiles phones, as they have been misused for personal communication with friends and family [39]. The above economic factors impose incremental costs of introduction of the technology that must be measured to determine cost-effectiveness of the mHealth program (*i.e.*, ratio of health gains benefits to costs).

### 2.3. Cultural

Cultural appropriation denotes the adoption of specific objects, ideas, or element of one culture by another culture. Within the context of mHealth, cultural appropriation refers to the use and meaning given to the mobile devices across different cultures, given its global availability [41]. For instance, in high-income countries, the mobile devices is perceived as a gadget; however, in low- and middle-income countries, it may be a status symbol of embracing global trends, ways of expressing social competence, and a means to strengthen local relations [41]. Social pressure or influence may explain, in part, the uptake of mobile phones against the backdrop of poverty [21,41]. For example, social obligations and codes have been established with the practice known as “flashing,” in which the caller lets the phone ring once or twice and then hangs up with the expectation the person will return the call and thus assume the cost of the call [41]. The use of technology by women is not acceptable in all cultures [37], and furthermore, women are less likely to own a mobile [21]. Mobile devices may influence family dynamics to maintain control of assets (*i.e.*, mobile phone) within the household [37]. Consequently, mHealth has the potential of aggravating financial problems and fostering social inequalities given cultural norms regarding women’s engagement with mobile technology.

### 2.4. Environmental and Physical

Energy demands may increase considerably as a result of no load power consumption, which occurs when the power charger is plugged in without having the mobile devices connected [42] or keeping mobile devices plugged in overnight rather than the few hours needed to recharge the batter. Energy consumption characteristics of mobile devices differ (e.g., 3G and GSM have higher energy demands) and are related to characteristics of the workload (*i.e.*, amount of processing required in a given time) [43]. Because of consumer demand, considerable emphasis is on developing energy-efficient and energy-saving devices and less on modifying behavior to conserve energy.

Electromagnetic radiation released by cell phone towers, or “dirty electricity,” is an environmental pollutant that impacts the balance of nature (e.g., decline in the number of house sparrows, extinction of bee colonies) [44], and with long-term exposure, may harm biological systems. Mobile devices also emit radiofrequency electromagnetic fields and absorbed mainly in the region of the brain where the phone is held [45]. Exposure to radiofrequency electromagnetic fields is highest in the temporal lobe on the side of the head the phone where it induces and promotes cancer through DNA damage [45,46]. Whether or not there is an association between digital mobile devices use and malignant brain tumor initiation (*i.e.*, carcinogenesis) and analogue and digital mobile devices use and promotion of cancer remains under debate as findings have not been consistent [47,48]. The newborn period is a time of significant growth; thus health hazards need to be considered as the radio frequencies have the potential to damage tissues and genes of the newborn [2]. The “Precautionary Principle”, that is measure to minimize exposure given uncertain risk, is recommended in instances where decades of exposure is anticipate (e.g., children) [47].

Mobile phones are made with precious and base metals (e.g., iron, nickel, copper, silver, gold). The growing demand for mobile phones together with their short lifetime (about 1 year) has repercussions for the environment and for health [49]. Recycling of mobile phones will be important to limit use of precious metals, while safe waste disposal strategies will be important to limit contamination, which can be a health hazard.

## 3. mHealth “Fingerprinting” Approach

The unintended harmful consequences discussed in this paper, namely psychosocial, economic, cultural, environmental and physical impact constitutes the framework [11] for the mHealth “Fingerprinting” approach. A mHealth fingerprinting approach can guide planning, implementation, and evaluation of mHealth interventions for maternal and newborn care. The mHealth fingerprinting approach can provide a logical framework for developing a comprehensive mHealth intervention strategy with appropriate indicators for success. As an evaluation framework, the mHealth fingerprinting approach can characterize, identify and quantify the association between mHealth and unintended harmful consequences [11]. A multi-disciplinary team of researchers will permit an examination of the dynamic nature of the interrelationship between psychosocial, social, and economic consequences and potential factors (e.g., lack of community engagement) contributing to unintended harmful consequences [11]. Transparency can be maintained by using the framework as a way of reporting outcomes including unintended harmful consequences [11].

## 4. Conclusions

Given that unintended harmful consequences of mHealth have not been adequately explored, whether mHealth improves maternal and newborn health outcomes remains uncertain—fuzzy logic—as the response is perhaps not true or false, but lies somewhere in between. The enterprise of mHealth and the accompanying demand to adopt mobile technology (e.g., surf internet, use health “apps”) needs to be systematically evaluated for their impact—positive, neutral or negative—not only on health outcomes but also on socio-ecological and environmental outcomes [2,50]. Health care providers and researchers need to monitor, with vigilance, the intended and unintended consequences of adopting mHealth for maternal and newborn care. A mHealth “fingerprinting” approach that monitors psychosocial, economic, cultural, environmental, and physical impacts provides a framework for researchers to evaluate mHealth interventions. Examining the effect of maternal and newborn mHealth on maternal-infant interactions is of paramount importance. Although this commentary focuses on the mother, extended family is integral in providing care in many societies around the world and influences the dynamic relationships between the infant and various members within the family. Thus, it is imperative to consider how mobile device uses in a household influences caregiver-infant interactions and interactions between family members.
